# On the Treatment of Puerperal Eclampsia

**Published:** 1871-07

**Authors:** Harvey Jewett

**Affiliations:** Canandaigua, N. Y.


BUFFALO

Medical and Surgical Journal.

VOL. X. JULY, 1871. No. 12.

### Original Communications.

ART. I.—*On the Treatment of Puerperal Eclampsia.* By HARVEY JEWETT, M. D., Canandaigua, N. Y. A paper read before the State Medical Society, at Albany, 1871.

Puerperal Eclampsia may be regarded as one of the most frightful and alarming diseases that medical men are called upon to treat.

In view of the new and tender relation of the young mother to her offspring as well as the anxious solicitation of relatives and friends, there rest no ordinary degree of responsibility upon the medical attendant who suddenly and unexpectedly finds his patient (who, a moment before was flushed with bright prospects and high hopes for the future) insensible, frightfully distorted with violent convulsions.

The suddenness of the attack—the alarming character of the spasms—the consternation of attendants and relatives—all contribute to make the case one of fearful magnitude and responsibility, and demands at the hands of the attending physician prompt and positive measures for relief.

Unless we are successful in arresting the spasms in a short time the patient is doomed to a speedy death from the repeated recurrence of the convulsions.

Hitherto—in my own experience at least—so large a proportion of cases have proved fatal when guided by the highest authority in the treatment, that I have been induced to cast about for some other theory, and more reliable remedial agents to arrest the spasms and save the patient. In this regard I am happy to say that in some measure I have been successful. At all events, when my patients were treated in strict accordance with the best authority laid down in the books—they died. Whereas, when treated upon the presumptive theory that the convulsions are the result of "Materies Morbi," generated during pregnacy, and that delivery is a developing influence of the peculiar morbid condition of the system, acting upon the brain and spinal nerves—or as Dr. Tyler Smith expresses it, as dependent upon some irritation of the excito-spinal nerves, they have recovered.

These views are founded upon Dr. Marshall Hall's researches into the Physiology of the Nervous System, and a practical application of that eminent writers discoveries to the pathology and treatment of Puerperal Eclampsia.

Many cases, apparently well authenticated, are on record, where females in the earlier stages of gestation, and without labor pains as a procuring cause, or when pregnancy does not even exist, have suffered from this peculiar form of Eclampsia. But these, I apprehend, may be regarded as exceptional cases, or more properly ruled out of the classification under discussion, and treated under the head of Hysteria or Epilepsy.

It is far from my purpose, however, in this short paper, to allude to the well authenticated classification of these cases, or to speak of that which all medical men can so easily refer to in any recognized authority on this subject. My object is merely to make a practical note on the treatment of this disease, which, to some extent, has bid defiance to the remedies so universally recommended by the highest authorities both past and present, and thus draw the attention of the profession to a plan of treatment and the use of remedies which have proved eminently satisfactory in my hands.

What is the nature and the cause of the disease we have to contend with? Is it the blood, or is it the brain or nervous system we have to deal with in our efforts to arrest the spasms? I apprehend neither exclusively, and both, to a certain extent, in their re lation to each other.

In all cases of pregnancy the blood and fluids of the system are abnormal—are changed, to a greater or less extent, from a healthy standard, and charged with certain influences calculated to overwhelm the nervous system, and tend to the developement of Eclampsia. Whether this toxic influence in different individuals is in degree or kind, is not well understood. We know that certain females are strongly predisposed to take on this spasmodic condition without any well defined indications preceding the attack of Eclampsia. This poisoned condition of the fluids of the system may arise from various causes, but principally from the pressure of the gravid uterus upon the renal veins. The pressure of any hard body upon the kidneys tends to interfere seriously with the function of that organ—to produce congestion, thereby abating its eliminating function, and producing toxenia of the blood. The non-elimination of the kidneys under any circumstances would necessarily produce more or less mischief, but especially so during pregnancy, when the blood requires an extra degree of depuration to cast off the excrementitious matter from the child and mother.

This faulty or impaired function of the kidneys is marked in the latter stages of gestation by extreme œdema of the lower extremities—a peculiar waxy puffiness of the face, scanty urinary secretions, almost invariably loaded with albumen, together with general lassitude, weariness of body and confusion of mind. I would not be understood to say, however, that when all these symptoms are prominently marked that Eclampsia necessarily follows, but that convulsions seldom occur without these primary indications, and especially albuminous urine.

According to Niemeyer's theory—these symptoms are due to a temporary parenchymatous change or degeneration of the kidney itself,and not to congestion or Nephritis from the pressure of the gravid uterus upon the emulgent veins,which we can readily understand,will cause both albuminous urine and the retention of a large amount of urea in the blood.

If the system is charged with toxic influences accumulated during gestation, which act as a procuring cause of the spasms, then it should be an object of vital importanoe to remove this agent or influence whatever it may be, as speedily as possible.

Almost all writers, for the last half century, have based their practice upon the theory that the brain in primiparous females, es pecially, is to be regarded "ipso facto," in a hyperæmic state for weeks and months preceding parturition. This was supposed to be consequent of the pressure upon the descending aorta—and also during violent and long continued labor pains, the solid contracted globe of the uterus is pressed violently against the descending aorta—thereby impeding the flow of the blood to the lower extremeties, and necessarily compelling the carotids and superior vessels to carry a preternatural quantity to the brain.

Based upon this theory of the pathology of the disease, we have no alternative but to carry out the advice so uniformly and peremptorily laid down in all works on this subject, both in this country and by continental writers—and that is to bleed copiously as the first and last great remedy—the extent to which it is carried being regulated by the violence and frequency of the convulsions, without much reference to the amount left in the system, absolutely essential to carry on the vital processes.

The history of the case—previous habits and peculiarities of the patient in regard to predisposition and exciting causes, are all ignored as indications for or against the use of the lancet, and we are commanded to bleed almost empyrically, to the utter exhaustion of the vital forces of the patient. The practitioner is left without any guiding principles, any data by which to regulate his practice in the use of this important and powerful remedy.

In compliance with this advice I bled, in my earlier practice, copiously, and repeatedly with very unsatisfying results, as in a large proportion of cases my patients died under the treatment; and simply because it did little or nothing towards removing the cause which produced the disease.

The introduction of chloroform as an anesthetic agent has been in this as in many other diseases, a boon of priceless value to suffering humanity. Not that I believe there is anything in the use of chloroform that tends in the least degree to remove the existing cause of the spasms—and I never administer it with any such view, but simply to abate the frequency and violence of the convulsions, and give time for eliminating remedies upon which we are to rely for a radical cure.

The three great channels upon which we are to depend for this purpose are the skin, the kidneys, and the bowels. The first two are too slow and indirect in their operation to be made available and answer the indications of the case, and we are to depend as the only alternative upon the *vicarious* action of the powels to cast off this poisonous element in the blood, and stay the progress of the spasms.

In all cases of convulsions I administer chloroform at once. If the patient has not been delivered and the head of the child is within reach of the forceps, apply them, and deliver as speedily as possible. If this is not practicable, and version can be effected, this is the next best plan of relieving the patient of this source of irritation. When this has been affected, give croton oil in connection with turpentine enemas in such quantity, and as frequently repeated as the stomach will tolerate, until the bowels have been freely evacuated.

In scarcely an instance under my observation has the patient had more than one or two convulsions after this has been effected, and generally they cease altogether, if the evacuation is copious and liquid in character.

Prior to 1860, I find ten recorded cases that were treated in compliance with the best authority of that time—by copious and repeated bleeding—cupping, leeches, blisters, sinapisms, cold to the head and spine, opium, anti-spasmodics, &c., &c. Of the ten cases thus treated, seven died.

Since that period I find six cases that were treated with chloroform and drastic cathartics, not one of the number were bled and all recovered.

There exists in Puerperal Eclampsia such a uniformity of symptoms and condition that I need give but two or three as representative cases, in illustration of my views concerning its treatment.

Case 1st.—Mrs. T—, aged 25, of delicate, nervous temperament, was delivered of her first child after an ordinary labor in July 1864. Just at the termination of labor, and before the removal of the placenta, she went into a violent convulsion. She was put immediately under the influence of chloroform and ether, and as soon as the spasm subsided, given three drops of croton oil in a tea-spoon full of milk. She was kept slightly etherized for an hour and a half, when she had another convulsion. The coma was quite profound after the second spasm, the breathing loud and stertorous. Two drachms of spirits of turpentine was beaten with the yolk of an egg diluted in a quart of warm water and used as an enema. In half an hour there was a profuse evacuation of the bowels, the chloroform was gradually withdrawn and in six or eight hours the patient was restored to consciousness and recovered in due time without any recurrence of the convulsions.

CASE 2d.—Mrs. F—, aged 23, of full, plethoric habit and sanguine, nervous temperament, was confined with the first child in March 1868. The labor was progressing satisfactorily, the head of the child was engaged in the lower straight of the pelvis, when suddeniy without any premonition, she went into a convulsion. As soon as practicable chloroform was administered—the forceps applied and the delivery effected. Three drops of croton oil were administered in milk. In half an hour it was evident the oil was taking effect from the internal disturbance. In one hour from the completion of the first she went into another spasm, from the effect of which she was insensible for several hours. The oil operated freely in an hour and a half after it was given, and the patient recovered in due time without any untoward symptoms.

CASE 3d.—Mrs. A—, aged 40, of delicate health and nervous temperament, was confined Dec. 1870, with her fourth child. The labor was completed in three or four hours without any unusual indications, except, she said there was pain in the head and glimmering before the eyes. In half an hour after the birth of the child and the removal of the placenta she remarked that she could not see, and was instantly seized with a convulsion. A combination of chloroform and ether was administered, and three drops of croton oil given. In an hour she took two drops more, and at the end of two hours she took two drops more, making in all seven drops of croton oil without any effect.

Large enemas containing turpentine were given a short intervals, which were retained. In adddition to the croton oil and enemas during the twelve hours, she took 3 oz. of castor oil, which had no effect on the bowels until the end of seventeen hours from the birth of the child, when the medicine operated freely as a cathartic. During this period the convulsions recurred at intervals of from one to two hours, making in all twelve convulsions in seventeen hours. After the operation of the cathartic the spasms ceased altogether, and in two or three days the patient gradually returned to consciousness. In this connection it would be proper to state that during the convulsive period, one drachm of bromide of potash was given, and during the last hour, immediately preceding the cessation of the convulsions, she took at three different times, eighty grains of chloral. It might be claimed, inasmuch as the convulsions ceased in connection with the administration of the chloral, that relief is properly due to that remedy. My own experience in the use of this agent in this disease is so limited, that I can arrive at no definite conclusion as to its power in controlling or mitigating its severity. This is an instructive case, and one from which different deductions may be drawn as to the credit due to the remedies administered.

In view of all the facts connected with the case, however, it is my opinion that little or no benefit was derived from the anæsthetics, bromide of potash or hydrate of chloral, inasmuch as there was an unmitigated persistence of the spasms until the action of the cathartic, when they ceased altogether.

During the seventeen hours the patient was kept almost constantly under the influence of chloroform and ether, which had the effect, together with the spasms, of producing severe capillary bronchitis, with laryngeal inflammation, thereby retarding convalescence of the patient, and at one time seriously threatened her life. The aphonia was complete, and lasted between two and three weeks.

In the three cases just cited, and in every case where tests were made, a large amount of albumen was found in the urine, and the specific gravity ranged about 1020.

The general principles applicable to the treatment of other forms of disease are eminently proper in the treatment of Puerperal Eclampsia.

If, when strictly carried out, they do not always tend to definite and satisfactory results, they will at least, reconcile the apparently contradictory and conflicting opinions and theories of various authors on this subject.

There is some diversity of opinion in reference to artificial interferenc in the progress of natural labor. Perhaps no arbitrary rules can be laid down to govern, under all circumstances, but it can be safely assumed that if delivery is practicable, either with forceps or by version, it is proper to affect it as speedily as is compatible with the surrounding indications. Upon no one point is there such universal unanimity of sentiment as that of copious and repeated bleedings to relieve the hyperemia of the brain, which, in a large proportion of cases, exists only in the fertile imagination of visionary theorists. In my own limited experience and observation, it has been perfectly impotent to arrest or even to mitigate the violence of the spasms.

Grave objections may be urged against the indiscriminate use of chloroform in the treatment of this disease. It can not have escaped the observation of any one, in the treatment of these cases, that the heart's action fluctuates in a remarkable manner. The pulse, at times, indicates a preternatural degree of vascular activity and force—at other times so feeble as scarcely to be felt, and may be suspended altogether if the effect of chloroform be super-added to the other depressing influences.

Then, again: if chloroform is free from this objection, we may very properly ask, how much does it accomplish in the way of holding the convulsions in check, and thereby give time for the radical eliminating remedies? The legitimate action of chloroform is confined to the function of the cerebral or sentient portion of the nervous system. It neither abolishes nor diminishes the proper function of the excito-motory or spinal nerves, as indicated in the suspension of suffering, but not in the least degree interfering in the progress of natural labor. If this be true, we may very naturally infer that cloroform has little or nothing to do with the arrest or suspension of these involuntary spasms, any more than it has to do with the arrest of natural labŏr-pains.

If puerperal eclampsia is the result of uremia or any other poison acting upon the excito-motory spinal nerves, as the result of non-elimination during the period of gestation, we are, in the treatment of this disease, to address our remedies to the casting off of this poison by the use of drastic cathartics, as the only channel we can make available in the exigencies of the case; and in no instance under my observation, when it has been faithfully carried out, has it failed to produce satisfactory results.

The obscurity hitherto connected with the remote and proximate cause of this disease has left prophylactic or preventive measures quite out of view. Scarcely any suggestions are on record in reference to preventive treatment.

In puerperal patients, where there is reason to apprehend the development of eclampsia, from the obscure indications in the case, I have been in the practice of giving saline diuretic remedies, such as sulphate of magnesia, acetate of potash, or the super-tartrate of potash with flowers of benzoin. How much benefit results from the use of prophylactic remedies is a question not easily solved, as we can have no assurance that the convulsions would have resulted had not the remedies been administered. Frequent tests for the presence of albumen should be instituted during the latter period of gestation, and the diuretics persevered in until the urine is comparatively free from the presence of albumen.

